# Poorer event-based compared to time-based prospective memory in a computerized household chores task

**DOI:** 10.1007/s00426-026-02338-x

**Published:** 2026-07-07

**Authors:** Shayne Loft, Ryan Li, Cathryn McKenzie, Romola S. Bucks, Steven P. Woods, Michael Weinborn

**Affiliations:** 1 School of Psychological Science, The University of Western Australia, Perth, WA 6009 Australia; 2 Applied Games and Simulations, Perth, Australia; 3 Department of Psychology, University of Houston, Houston, TX USA

**Keywords:** Prospective memory, Time-based, Event-based, Memory for intentions screening test, Attention

## Abstract

Remembering to carry out deferred tasks in the future is referred to as Prospective Memory (PM). In everyday life, PM can often require switching attention between environmental stimuli, but traditional laboratory PM tasks do not necessarily capture this task element. The aim of the current study was to compare event- and time-based PM in a House PM task, designed to capture this component of PM by requiring individuals to remember to complete multiple housework tasks. Participants completed the House PM task and the Memory for Intentions Screening Test (MIST). In Experiment 1, participants were undergraduate students or individuals from the local community. The purpose of Experiment 2 was to replicate the outcomes of Experiment 1 and to test for generalizability to a sub-sample of older adults. Across two experiments, we replicated prior findings in the literature of poorer time- compared to event-based PM accuracy on the MIST. In contrast, in both experiments, time-based PM accuracy was better than event-based PM accuracy in the House PM task. In the MIST, event-based PM cues are directly presented within ongoing task activity and are thus in the focus of attention. In contrast, in House PM, individuals need to remember to periodically switch attention between household rooms to detect event-based cues that are perceptually peripheral to ongoing task activity. These outcomes are important because PM demands in everyday life and work settings can require individuals to not only remember to perform a deferred task, but also to periodically remember to switch attention.

## Public significance statement

The ability to remember to perform deferred actions in the future is critical in everyday life and work settings. In a task designed to represent prospective memory requirements that individuals may encounter in a common household, we demonstrate that contrary to the extant prospective memory literature, event-based prospective memory can in fact be poorer than time-based prospective memory due to attention switching requirements.

Prospective memory (PM) refers to the ability to remember to perform intended actions at an appropriate moment in the future (Einstein & McDaniel, 1990; Rummel & McDaniel, 2019). Event-based PM requires individuals to execute a deferred action upon encountering a target event (cue). Time-based PM requires individuals to perform tasks at a specific time, or within some time interval. PM functioning plays an important role in independent living (Woods et al., 2012) and is critical in modern work environments such as healthcare and aviation (Dismukes, 2012; Loft, 2014; Loft et al., 2019).

Traditionally, PM has been measured in laboratory settings (Einstein & McDaniel, 1990) with abstract tasks requiring completion of a single or repeated PM action while performing an ongoing task, in which task stimuli are centrally presented on a display and thus attention does not need to shift between competing task stimuli. This does not necessarily reflect the complexities of PM in everyday life, which often entails the need to shift attention between environmental stimuli to complete PM tasks (Dismukes, 2010, 2012). PM can be measured through tests containing more naturalistic tasks, but with substantially limited experimental control (Rummel & Kvavilashvili, 2019). There are also self-report PM measures that provide insight into perceived PM ability, though these are subject to self-report biases (Uttl & Kibreab, 2011). Several research groups have developed virtual reality (VR) PM paradigms (e.g., Barnett & Coldiron, 2023; Mathews et al., 2016; Kourtesis & MacPherson, 2023). VR paradigms are designed to retain the experimental control required to make psychological inference, but to be more representative of everyday PM demands because they require attention switching. For example, Barnett and Coldiron (2023) required participants to complete deferred tasks in a virtual kitchen and reported poorer PM for healthy older- compared to young-adults, and for older adults with clinical conditions compared to healthy older adults. Mathews et al. (2016) used a VR house task to train stroke survivors to complete PM tasks, and that training improved subsequent PM functioning. To our knowledge however, no prior VR study has systematically compared event-based PM to time-based PM.

In the current paper, we introduce a computerised *House PM task*. The House PM task requires individuals to complete an ongoing task while remembering to perform tasks relating to household activities that require switching attention between environmental stimuli. Across the two experiments, the focus was on the comparison of event- compared to time-based PM. In Experiment 1, we also manipulated the difficulty of the ongoing task, and, to establish the generalisability of outcomes across age, Experiment 2 included older as well as younger adults. In both experiments, as a comparison point, we assessed and compared event- to time-based PM in a well-validated clinical laboratory-based measure of PM (the Memory for Intentions Screening Test; MIST; Raskin, 2009).

## Event- vs. time-based prospective memory

Einstein and McDaniel (1990) developed a laboratory PM paradigm to emulate conditions in which people, who are concurrently engaged in an ongoing task, are required to remember to execute earlier established intentions. Variants of this paradigm has been used in many PM studies (see Rummel & McDaniel, 2019, for review). The most common ongoing task is lexical decision making, which requires individuals to indicate (via the keyboard) whether strings of presented letters constitute a real word or not. Participants may also need to remember to press an alternative PM response key if a word presented on the display is an ‘animal’ word (e.g., tiger) (event-based PM), or at a specific time (time-based PM). Participants are not reminded of the PM task during completion of the ongoing task and are, therefore, required to remember to execute the intended action in response to PM targets or at a certain time.

The Einstein and McDaniel (1990) paradigm has been used extensively to test event-based PM by manipulating PM cue features and the ongoing task in which they are embedded (e.g., Einstein et al., 2005; Hicks et al., 2005; Smith, 2003; Strickland et al., 2018). Comparatively fewer studies have examined time-based PM using this paradigm (e.g., Conte & McBride, 2018; Huang et al., 2014). Nonetheless, across variants of the Einstein and McDaniel paradigm, time-based PM accuracy is typically poorer than event-based PM accuracy (e.g., Conte & McBride, 2018; Einstein et al., 1995; Kliegel et al., 2001; Park et al., 1997) or at least equated to event-based PM accuracy (Jäger & Kliegel, 2008). An alternative and well-validated laboratory-based measure of PM, the MIST (Raskin, 2009; Woods et al., 2008), requires individuals to remember to perform several concurrently held time- and event-based PM tasks while completing an ongoing task puzzle. On the MIST, time-based PM accuracy is typically poorer than event-based PM accuracy in both healthy adults and clinical populations (e.g., Doyle et al., 2013; Kamat et al., 2014; Palermo et al., 2016, 2020; Saha & Christman, 2014; Woods et al., 2008).

Experiments using the Einstein and McDaniel (1990) paradigm (and the MIST), and resulting theory development, have substantially increased our understanding of PM processes (see Rummel & Kvavilashvili, 2023, for review). The current theoretical consensus in the PM literature is that time-based PM is more difficult than event-based PM in these PM paradigms because time-based PM relies to a greater degree on self-initiated memory retrieval, whereas event-based PM retrieval is cued by the external presentation of events (Rummel & Kvavilashvili, 2023). Presentation of external events, particularly when they overlap with ongoing task stimulus processing or are otherwise made distinctive, can spontaneously elicit event-based PM retrieval with little to no cognitive capacity required (Einstein et al., 2005; Scullin et al., 2010; but see Smith, 2003). In contrast, time-based PM tasks require individuals internally to monitor time (prospective timing estimation; Zakay & Block, 1996), or to maintain the intention to periodically check a peripherally placed clock to externalise prospective timing estimates (Huang et al., 2014), both of which are highly capacity-consuming (Waldum & Sahakyan, 2013), particularly the former. However, as discussed further below, the relevance of these theoretical assertions to PM paradigms that require attention to be shifted between environmental (task) stimuli is unclear.

### Experiment 1

The House PM task requires individuals to remember to complete event-based (e.g., “empty the washing machine in the bathroom when the light turns green”) and time-based (e.g., stir the pot of soup in the kitchen in 5 minutes”) PM tasks in various rooms, such as the bathroom or kitchen respectively, while completing an ongoing bill calculation (arithmetic) task in a different room (e.g., home office). We also manipulated the difficulty of the ongoing task (i.e., whether a calculator could be used for bills) to test the sensitivity of the House PM paradigm to a well-established finding in the PM literature. A meta-analysis by Matos et al. (2020) indicated individuals are less likely to remember to perform PM tasks under conditions in which ongoing tasks increase working memory and/or executive control load and, on this basis, we predicted poorer PM accuracy in more difficult ongoing task conditions in the House PM task.

**Fig. 1:**
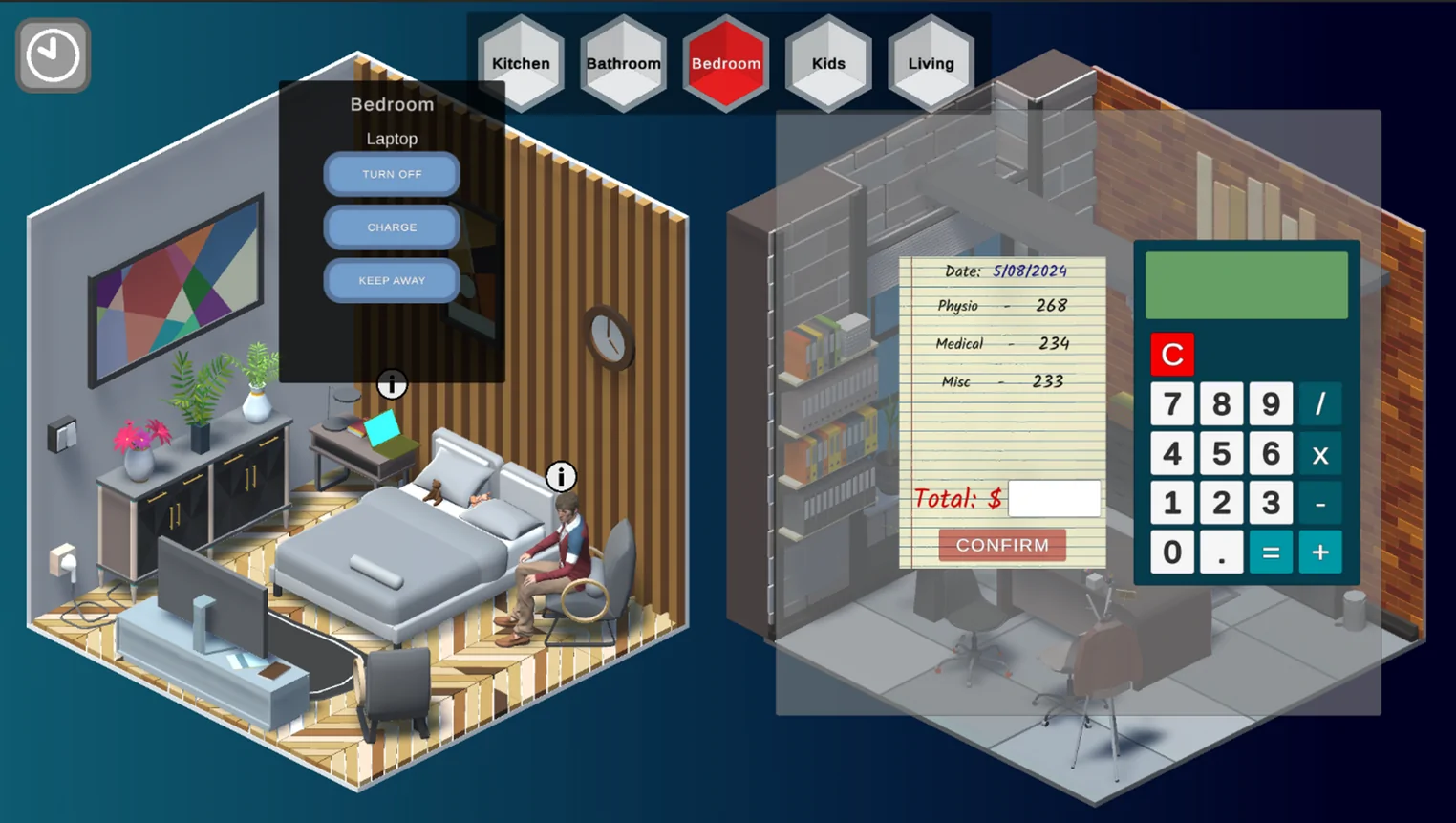
Low ongoing task condition: Bill calculation questions were completed using the calculator. In this example, participants were required to select the ‘bedroom’ and use the ‘laptop’ to select the correct PM task action from a drop-down list that included two distractors. Clock in top left-hand corner if selected displayed the current time

Contrary to theory and empirical findings in the PM literature, we made the novel prediction that event-based PM accuracy would be poorer than time-based PM accuracy (and potentially more so at higher ongoing task difficulty). Event-based cues in the House PM task are inherently more *non-focal* than the non-focal PM cues used in the MIST or in the Einstein and McDaniel (1990) paradigm. PM cues are more non-focal to ongoing tasks when there is lower overlap between ongoing task processing and the information that needs to be processed to detect PM cues (Einstein et al., 2005; Strickland et al., 2018). Notably however, in the Einstein & McDaniel and MIST paradigms, event-based non-focal PM cues are nevertheless directly presented within the focus of ongoing task activity (e.g., presentation of a red pen to the participants by an experimenter during the MIST, PM cues embedded in letter strings centred on a computer display) and thus are in the focus of visual attention (Einstein et al., 2005; Scullin et al., 2010). Time-based PM is thus more difficult in these paradigms because they require prospective timing estimates (i.e., checking the clock). In contrast, in the House PM task, individuals are required to remember periodically to switch attention between household rooms to monitor for event-based PM cues that are visually peripheral to ongoing task activity (as illustrated in Fig. 1, participants can enter different rooms by selecting room icons). In this manner, event-based PM in the House PM task requires individuals to instantiate and maintain a time-based plan for checking different rooms to check for the potential onset of event based-cues and thus requires *both* self-initiated time- and event-based PM monitoring (Dismukes, 2010).

In the House PM task, we presented a clock icon on the top of the display that, when selected, indicated the current time (emulating a household clock). We would, however, expect the time monitoring demand (prospective timing estimation; Zakay & Block, 1996) associated with event-based PM attention switching requirements to be greater than time-based PM attention switching requirements in the House PM task, because event-based PM cues can be presented at any point during the PM retention interval and are not, therefore, well-scaffolded by the passage of time (Huang et al., 2014; Waldum & Sahakyan, 2013). Thus, we predicted poorer event- compared to time-based PM accuracy on the House PM task, but the opposite to be the case for the MIST (replicating the prior MIST literature).

## Method

### Participants

Participants were recruited from the University of Western Australia (UWA) undergraduate research participation system, and they received two study credits for participation. Participants were also recruited from the UWA community participation system and received reimbursement of $15 AUD per hour. Participants were tested individually in a two-hour face-to-face session. As this is the first study to have used the House PM task, we had no a priori effect size on which to base power calculations, thus we based our sample size based on obtaining a medium (i.e., theoretically meaningful) effect-size at a power of 0.99 (requiring 76 participants). Seventy-nine participants completed the experiment (62.0% female, 35.4% male, 2.6% unspecified). Age ranged from 18 to 49 years (*M* = 21.77, *SD* = 5.91). This research complied with the National Statement of Ethical Conduct in Human Research and was approved by the Human Research Ethics Office at UWA (2022/ET000228). The procedures used adhere to the tenets of the Declaration of Helsinki. Informed consent was obtained from each participant.

### Design

A 2 × 2 within-subjects design was used, with two levels of ongoing task difficulty (low, high), and PM cue type (event, time-based). Participants completed two house PM task sets, at low or high ongoing task difficulty, and each set contained two event- and two time-based PM cues. Participants also completed the MIST (only the PM type manipulated).

### Materials

#### The house PM task

The House PM task is a computerised task with two 20-min blocks. Participants complete an ongoing bill calculation task. Bill calculation required the addition of three randomly generated three-digit numbers (e.g., 222 + 266 + 218). In one block, participants were permitted to use a calculator (low ongoing task difficulty), whereas in the other block participants were not (high ongoing task difficulty). Figure 1 illustrates the low ongoing task difficulty condition where participants could use the calculator.

During each block, participants were presented with four PM cues; two event- and two time-based. The PM cues for Blocks A and B are presented in Table 1. Participants held a maximum of two PM intentions at any point and the retention intervals for PM tasks were equated across PM cue type. The order in which participants completed the two blocks was counterbalanced, and the block that provided the calculator was counterbalanced.

**Table 1:** PM task blocks A and B: Task type, cues, expected PM performance times, and associated PM retention intervals

Block	Task Type	Task	Item	Operation	Cue	Location	Expected performance time	Notification time	Retention interval
A	Time	Stir soup in the pot	Pot	Stir soup	N/A	Kitchen	0:09:00	0:01:00	0:08:00
A	Event	Remove clothes from the washing machine	Washing machine	Remove clothes	The washing machine light turns purple	Bathroom	0:10:50	0:03:50	0:07:00
A	Event	Refrigerate jelly on the table	Jelly	Refrigerate jelly	The jelly turns blue	Kitchen	0:17:15	0:13:15	0:04:00
A	Time	Remind Tom of coffee	Tom	Coffee is ready	N/A	Bedroom	0:19:00	0:16:00	0:03:00
B	Event	Charge the laptop	Laptop	Charge	Laptop’s screen colour turns red	Bedroom	0:11:05	0:01:05	0:10:00
B	Time	Make medical appointment with the phone	Phone	Made medical appointment	N/A	Living room	0:12:00	0:06:00	0:06:00
B	Time	Water the plant	Plant	Water	N/A	Bathroom	0:22:00	0:13:00	0:09:00
B	Event	Ask Tom to record TV program	Tom	Record TV program	Tom appears in the living room	Living room	0:19:20	0:14:20	0:05:00

The House PM task presented individual rooms on the display adjacent to the ongoing task room (office) used for the bill calculation task. As shown in Fig. 1, participants could move between rooms by selecting the room icon buttons at the top of the display, the selected room was then displayed on the left of the ‘office’, such that participants could switch attention between the PM relevant room and the ongoing task room in which they were making the household bill calculations. A clock icon in the top left-hand corner of the screen was available for participants to click to view the current time.

**Fig. 2:**
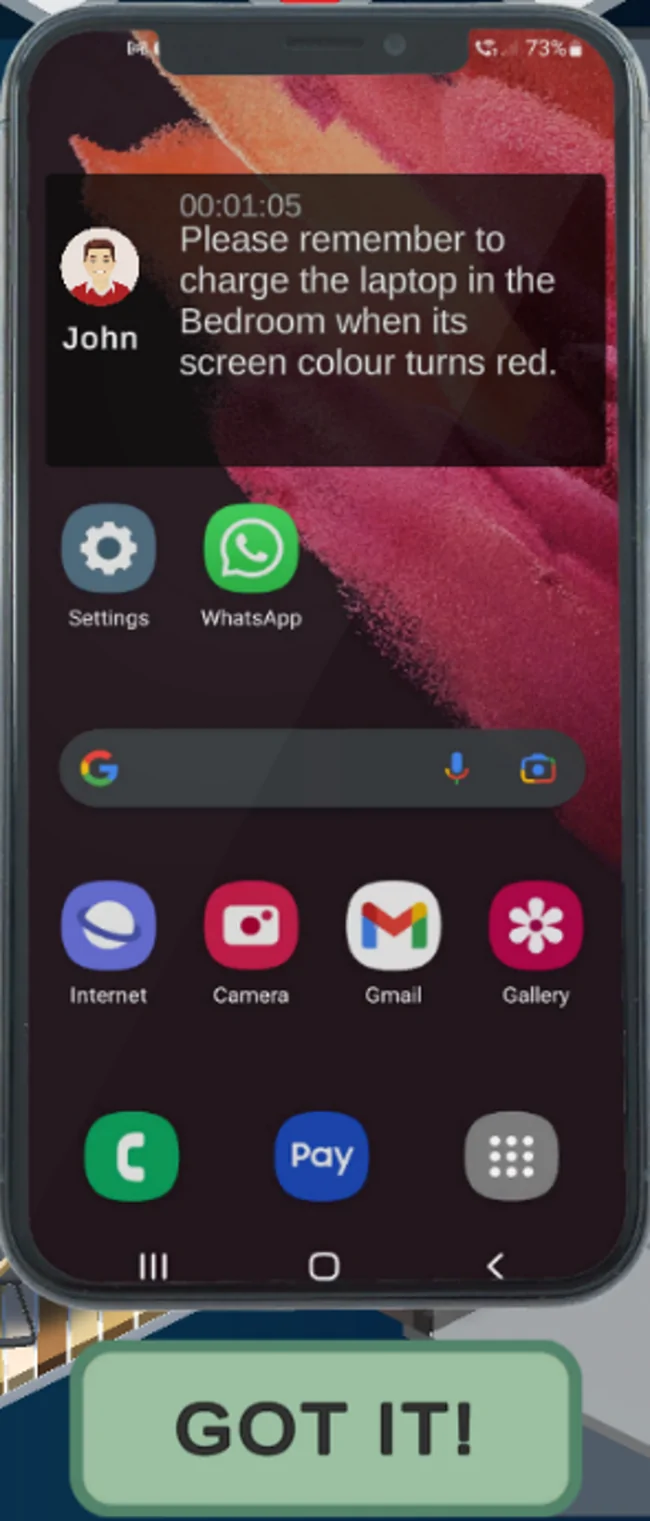
An example EB PM Task instruction presented as a text on a phone. Participants selected ‘GOT IT!’ to confirm that they had read the instruction

Participants received instructions for PM tasks through texts displayed on a phone icon (Fig. 2). Participants selected the ‘GOT IT’ button to confirm. Each room contained several interactive objects. For example, in the bedroom, there was a laptop that participants used to complete PM tasks. Once an object was selected, participants needed to select the correct task to perform. These tasks appeared in a drop-down list; and included the correct PM action (‘turn off’) and two distractors (‘charge’ and ‘keep away’).

Performance on the PM task was scored as correct if the correct PM action was performed within 20 s after the PM cue was presented (event-based), or within 10 s before or after the PM time (time-based). PM performance is expressed as the proportion of PM cues with correct responses.

At the conclusion of each block, a recognition memory test consisting of four questions required participants to identify the PM tasks that they had been asked to complete. For each PM task, the participant had to choose the correct answer from four options.

#### The memory for intentions screening test (MIST)

The MIST ongoing task consisted of word-search puzzles. The examiner instructed participants to complete eight PM tasks, four event- and four time-based. Event-based tasks were either verbal, for example “when I show you my phone tell me to put it on silent,” or actions, for example “when I show you a red pen, sign your name.” Time-based tasks also included verbal tasks, such as “in fifteen minutes tell me it’s time to take a break,” and action tasks, such as “in two minutes, ask me what time this session ends today”.

The MIST is commonly scored by awarding participants one point for responding at the correct time (time-based) or to the appropriate cue (event-based), and one point for providing the correct response (Sullivan et al., 2018). We amended this scoring to align with the House PM task by omitting MIST partial scoring. MIST PM performance was expressed as the proportion of PM cues correctly responded to within 1 min on each side of the cue time/cue presentation for PM tasks with a 2-minute retention interval, and within 2 min on each side for PM tasks with a 15-minute retention interval (see Woods et al., 2008).

The MIST retrospective recognition questionnaire consisted of eight multiple choice questions where participants identified the PM tasks they were required to complete. A maximum score of eight was possible for the MIST recognition test.

### Procedure

After providing consent, participants were presented with either the MIST or House PM tasks (counterbalanced order). Instructions for the House PM task were embedded in the task and informed participants how to calculate bills and move through rooms/interact with objects. Participants were informed that PM task instructions would be presented through a displayed phone icon, and that a clock was available to click to monitor time. Participants were instructed to work through the household bills as quickly as possible and to remember to complete the PM tasks. Each House PM task block had a duration of 24 min and contained four PM tasks. Participants then completed the House PM task recognition memory test. They were then given a one-minute break. The second House PM task block was then completed followed by the second recognition test.

Instructions for the MIST were administered verbally to the participant by an examiner who sat directly opposite, laying out all items needed to perform word-search puzzles (ongoing task) and PM tasks on a desk in front of participants. A clock was available to participants to check the time, located behind the participants left shoulder. The MIST duration was 26 min. Then participants completed the MIST recognition questionnaire.

## Results

Effect sizes for *F* tests were estimated with partial eta squared (small = 0.01, medium = 0.06, large = 0.14). Cohen’s (1988) *d* was used to estimate effect sizes for *t* tests (small = 0.20, medium = 0.50, large = 0.80). Small, medium, and large correlation effect sizes are 0.10, 0.30, and 0.50, respectively (Cohen, 1992). Descriptive data are presented in Table 2.

**Table 2:** Descriptives for the experiment 1: Mean (SD)

Variable			
*House PM task*		Low ongoing task difficulty	High ongoing task difficulty
Event-Based PM (proportion correct)	**0.65 (0.35)**	0.67 (0.34)	0.63 (0.36)
Time-Based PM (proportion correct)	**0.77 (0.31)**	0.79 (0.31)	0.76 (0.31)
		**0.73 (0.33)**	**0.70 (0.34)**
Recognition (proportion correct)		0.89 (0.14)	0.88 (0.16)
Ongoing Task (# bills correctly calculated) Clock Checking (Time-Based # clock checks)		74.61 (16.47) 19.6 (6.0)	58.27 (22.04) 19.5 (7.9)
*MIST* Event-Based PM (proportion correct) Time-Based PM (proportion correct) Recognition (proportion correct) Clock Checking (Time-Based # clock checks)	0.95 (0.12) 0.78 (0.22) 0.95 (0.07) 21.4 (6.6)		

MIST = Memory for Intentions Screening Test. MIST Clock Check *N* = 46). Marginal means presented in bold

### House PM task

#### Prospective memory accuracy

**Fig. 3:**
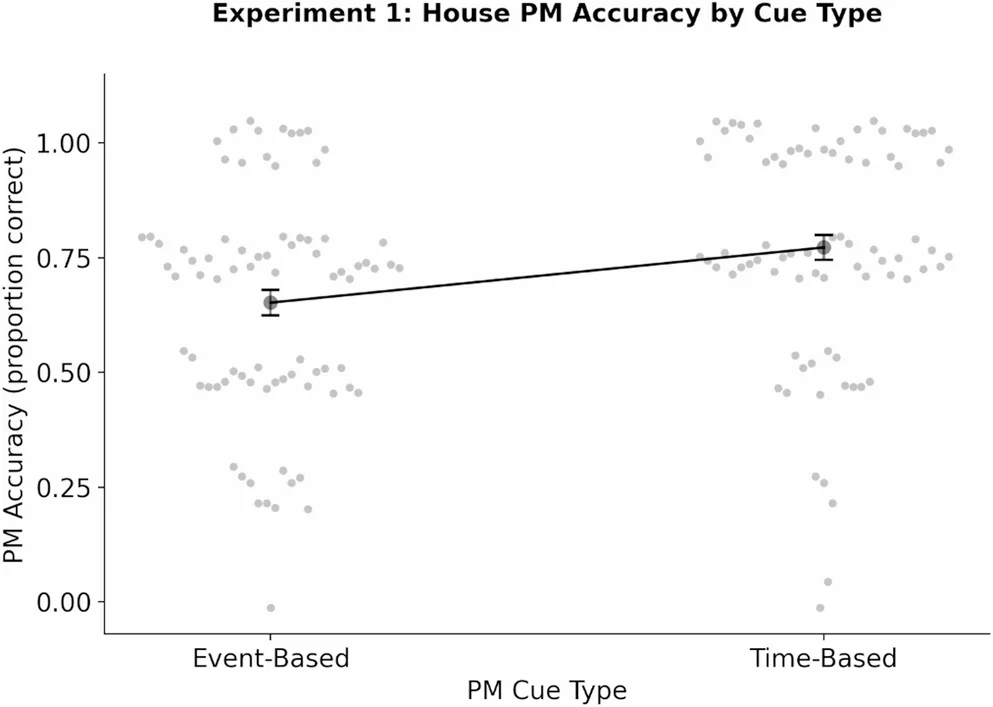
House PM accuracy as a function of PM cue type. The jittering of individual data points is required because there are only five possible data points for PM accuracy (0, 0.25, 0.50, 0.75 and 1)

A 2 (PM cue: event-based, time-based) x 2 (Ongoing task: high, low difficulty) repeated measures ANOVA was conducted on PM accuracy (Fig. 3). There was no difference in PM accuracy when ongoing task difficulty was high compared to low, *F* < 1. As predicted, time-based PM accuracy was better than event-based PM accuracy, *F*(1,78) = 11.16, *p* = .001, *η*_*p*_^*2*^ = 0.13. There was no interaction between ongoing task difficulty and PM cue type, *F* < 1. There was no difference for recognition test performance following the low compared to the high ongoing task difficulty block, *t* < 1, or for time-based compared to event-based PM, indicating that time-based versus event-based PM accuracy differences are not explained by differential failure to remember PM instructions.

#### Ongoing task performance

Ongoing task performance was defined as the number of correct bill calculations completed and was higher in low compared to high ongoing task difficulty blocks, *t*(78) = 6.02, *p* < .001, *d* = 0.68, indicating the manipulation was successful.

#### Clock checking

There was no difference in clock checking frequency in low compared to high difficulty ongoing task blocks, *t* < 1. In the low-, *r*(77) = 0.43, *p* < .001 (medium-to-large effect size), and high-difficulty blocks, *r*(77) = 0.29, *p* < .01 (medium effect size), time-based PM accuracy was positively correlated with clock checking frequency.

### Memory for intentions screening test (MIST)

**Fig. 4:**
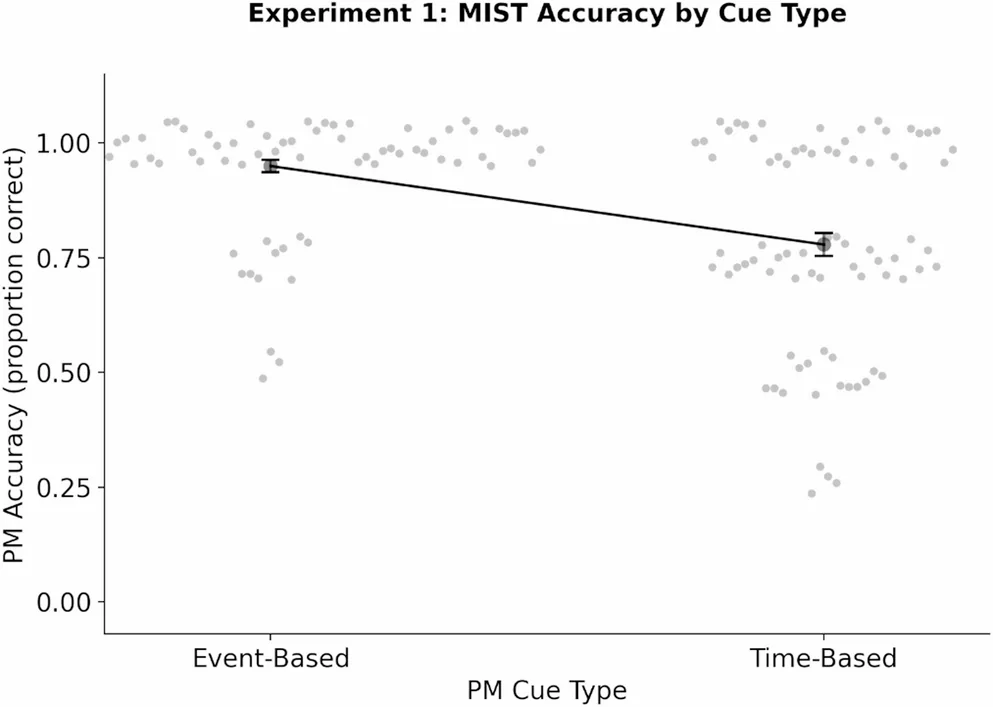
MIST PM accuracy as a function of PM cue type. The jittering of individual data points is required because there are only five possible data points for PM accuracy (0, 0.25, 0.50, 0.75 and 1)

As predicted, and in contrast to the House PM task, event-based PM accuracy was better than time-based PM accuracy for the MIST, *t*(78) = 7.10, *p* < .001, *d* = 0.96 (Fig. 4). Due to experimenter error, clock checking was not recorded for 33 participants. For the remaining 46 participants, clock checking frequency did not correlate with time-based PM accuracy, *r*(44) = 0.07, *p* = .66.

### Experiment 2

The primary aim of Experiment 2 was to test if the finding of superior time- compared to event-based PM accuracy in the House PM task would replicate. To establish the generalizability of outcomes across age, we included a sub-sample of older adults. Older adults typically perform more poorly than younger adults in laboratory PM tasks (Henry et al., 2004), and on the MIST (Kamat et al., 2014). We expected to replicate that here on the House PM task and the MIST, but as detailed further below we had low statistical power to detect age (group level) effects, and age-differences in PM accuracy were not the focus here. Rather, the focus of Experiment 2 concerned replicating the Experiment 1 outcomes, with higher PM accuracy for time- compared to event-based PM tasks expected for the House PM task, with the opposite finding expected to be replicated for the MIST.

## Method

### Participants

Younger participants were recruited from the UWA undergraduate participant pool and received course credit (*N* = 20, *M* = 21.4yrs, *SD* = 6.7yrs, *Range* = 18 to 48yrs, 80% female, 20% male). Older participants were recruited from the UWA Healthy Ageing Research Program and received a reimbursement of $15 AUD per hour (*N* = 25, *M* = 76.8, *SD* = 9.33 *Range* = 65 to 95 yrs, 88% female, 12% male). Participants were tested individually in a two-hour face-to-face session. With 45 participants, we had power of 0.99 to detect the large size effect (*η*_*p*_^*2*^ = 0.13) of PM cue type on House PM task accuracy found in Experiment 1. In contrast, we had relatively low power (0.74) to detect a large-sized effect of age on PM accuracy.

### Design

Experiment 2 used a 2 × 2 mixed-subjects design, with age (younger, older) as the between-subjects factor and PM cue (event-based, time-based) as the within-subjects factor.

### Materials and procedure

The material and procedure were identical to Experiment 1, other than the House PM ongoing task difficulty not being manipulated, with both House PM blocks providing a calculator for the ongoing bill calculation task.

## Results

The descriptive data are presented in Table 3.

**Table 3:** Descriptives for the experiment 2 sample; mean (SD)

Variable			
*House PM task*		Younger Adults	Older Adults
Event-Based PM (proportion correct)	**0.39 (0.32)**	0.61 (0.26)	0.22 (0.25)
Time-Based PM (proportion correct)	**0.52 (0.36)**	0.78 (0.23)	0.32 (0.33)
		**0.69 (0.26)**	**0.27 (0.29)**
Recognition		0.89 (0.12)	0.76 (0.12)
Ongoing Task (# bills correctly calculated)		86.12 (19.11)	41.62 (14.51)
Clock Checking (Time-Based # clock checks)		18.1 (6.1)	11.2 (5.4)
*MIST*			
Event-Based PM (proportion correct)	**0.90 (0.17)**	0.96 (0.09)	0.85 (0.20)
Time-Based PM (proportion correct)	**0.74 (0.20)**	0.88 (0.13)	0.63 (0.17)
		**0.92 (0.12)**	**0.74 (0.22)**
Recognition (proportion correct)		0.96 (0.07)	0.86 (0.10)
Clock Checking (Time-Based # clock checks)		19.9 (4.7)	13.9 (5.3)

MIST = Memory for Intentions Screening Test. Marginal means presented in **bold**

### House PM task

#### Prospective memory accuracy

**Fig. 5:**
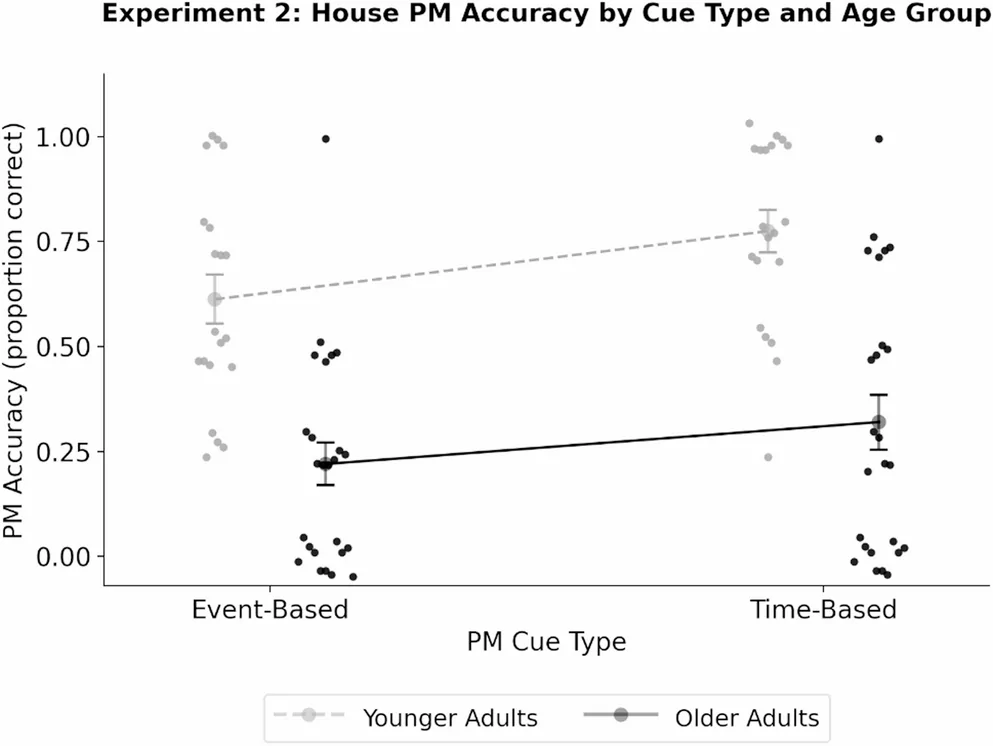
House PM accuracy as a function of PM cue type and age. The jittering of individual data points was required because there are only five possible data points for PM accuracy (0, 0.25, 0.50, 0.75 and 1)

A 2 (PM cue: event- vs. time-based) x 2 (Age: older vs. younger adults) mixed ANOVA was conducted (Fig. 5). As predicted, participants performed better on time- compared to event-based tasks, *F*(1,43) = 6.23, *p* = .02, *η*_*p*_^*2*^= 0.12, replicating Study 1. Older adults had poorer PM accuracy than younger adults, *F*(1,43) = 45.64, *p* < .001, *η*_*p*_^*2*^ = 0.52. There was no interaction between age and PM cue type, *F* < 1, indicating that the event- versus time-based PM accuracy difference was approximately equated across the two age-groups. However, follow up contrasts indicated statistically significant poorer event- versus time-based PM accuracy for younger (17% decline), *t*(19) = 2.46, *p =* .02, *d* = 0.66, but not for older adults (10% decline), *t*(24) = 1.29, *p =* .21.

Older adults had poorer House PM recognition test performance than younger adults, *t*(43) = 3.54, *d* = 1.08.

#### Ongoing task performance

House PM ongoing task performance was poorer for older compared to younger adults, *t(*43) = 8.88, *p* < .001, *d* = 2.62.

#### Clock checking

Older adults checked the clock less often than younger adults, *t*(43) = 4.03, *p*< .001, *d* = 1.20. For older adults, clock checking was correlated with time-based PM accuracy *r*(23) =0.66, *p* < .001, though not for younger adults, *r*(18) = 0.26, *p* = .27.

### Memory for intentions screening test (MIST)

**Fig. 6:**
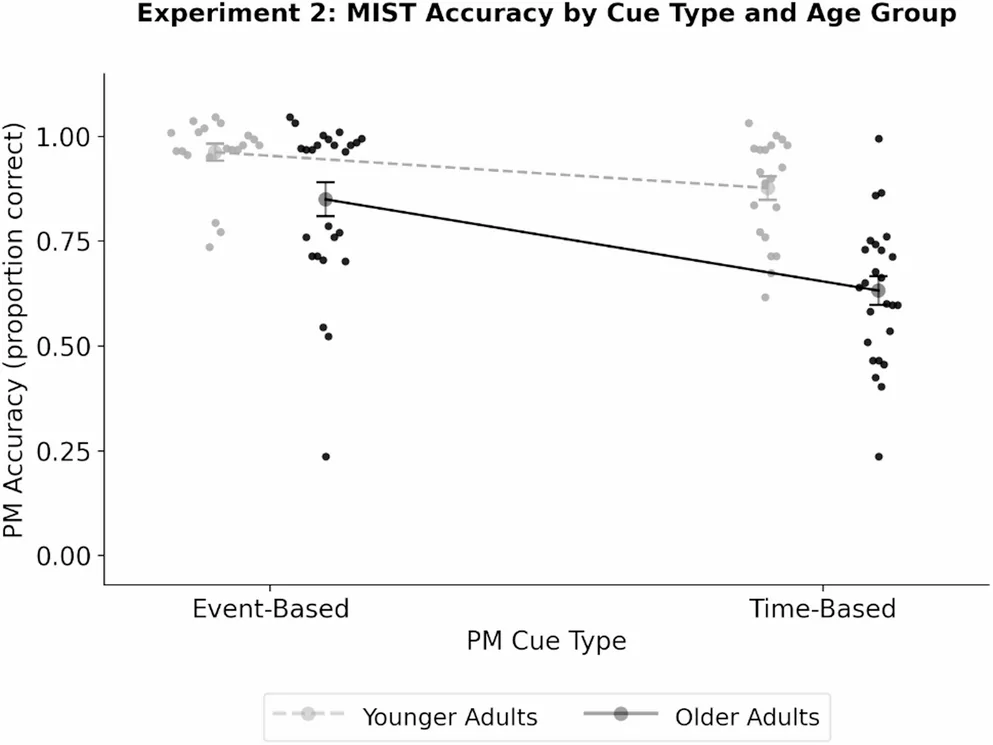
MIST PM accuracy as a function of PM cue type and age. The jittering of individual data points was required because there are only five possible data points for PM accuracy (0, 0.25, 0.50, 0.75 and 1)

As predicted, event-based PM accuracy was better than time-based PM accuracy, *F*(1,43) = 42.05, *p* < .01, *η*_*p*_^*2*^= 0.49, replicating Experiment 1 (Fig. 6). Older adults had poorer MIST PM accuracy than younger adults, *F*(1,43) = 18.65, *p*<.001, *η*_*p*_^*2*^=0.30. There was an interaction between age and PM cue type, *F*(1,43) = 7.801, *p* = .01, *ηp 2* = 0.15, with the effect of age on PM accuracy greater for time-based compared to event-based PM tasks. Follow up contrasts indicated higher PM accuracy for time- compared to event-based PM for both younger, *t*(19) = 2.10, *p* = .04, *d* = 0.78, and older, *t*(43) = 5.32, *p* < .001, *d* = 1.19. Older adults’ recognition test performance was poorer than young adults, *t*(24) = 8.37, *p*<. 001, *d* = 1.16.

Older adults checked the clock less than younger adults, *t*(43) = 3.91, *p*<.001, *d* = 1.18. For older adults, the correlation between clock checking and time-based PM accuracy was not significant, *r*(23) = 0.39, *p* = .050. For younger adults, the clock checking also did not correlate with time-based PM task accuracy, *r*(18) = 0.08, *p*= .74.

## Discussion

PM is critical for independent living (Rummel & Kvavilashvili, 2019; Woods et al., 2012) and in modern work settings (Dismukes, 2012; Loft, 2014). Traditionally, PM has been measured with abstract tasks consisting of a single repeated PM task requirement, and in which individuals do not need to remember to shift attention between peripherally separated task stimuli. This does not adequately reflect the complexities of everyday PM (Dismukes, 2010, 2012; Rummel & Kvavilashvili, 2019). The current study tested a new House PM task designed to be more representative of everyday PM by requiring individuals to complete an ongoing task while remembering to shift attention across a household to perform several delayed, housework related tasks.

Before moving to the main findings of interest, in Experiment 1, the difficulty of the household bill calculation task did not impact PM accuracy, a finding inconsistent with prior PM research (Matos et al., 2020). Further research is required, but a potential explanation is that participants were more “immersed” in the lower- compared to higher-difficulty ongoing task due to the ease of completing household bill calculations using the calculator, which may have counteracted any impact that the higher difficulty ongoing task had on participant executive load/working memory demands. Alternatively, the ongoing difficulty manipulation, despite impacting ongoing task performance, may not have been strong enough to impact the capacity of participants to complete the PM task (Hicks et al., 2005; Smith, 2003). Older adults typically perform PM tasks less well than younger adults in the laboratory (Henry et al., 2004), and we replicated that in Experiment 2, albeit age differences were not our focus. For time-based PM in the House PM and the MIST, older adults made fewer clock-checks than younger adults, possibly due to age-related declines in the prefrontal regions responsible for executive function (Henry et al., 2004). Future research can use the House PM task to examine potential differences between event- and time-based PM functioning in older adults and clinical populations.

The central finding of interest was that, replicated across the two experiments, event-based PM accuracy was poorer than time-based PM accuracy on the House PM task, with the opposite effect for the MIST (the latter finding being consistent with prior MIST research; e.g., Doyle et al., 2013; Kamat et al., 2014; Palermo et al., 2016, 2020; Saha & Christman, 2014; Woods et al., 2008). In contrast to the MIST, and other paradigms, such as that of Einstein and McDaniel (1990), the House PM task requires participants to remember to switch attention between rooms to monitor for potential event-based PM cues that are presented peripherally to ongoing tasks. Detecting a specific PM cue word (“cat”) in the Einstein and McDaniel paradigm is more focal to ongoing lexical decision than detecting any “animal word’ (Einstein et al., 2005; Scullin et al., 2010), but notably, in both cases, the PM cue is directly embedded within ongoing task stimuli centred on the display and thus is inside the focus of visual attention. Similarly, in the MIST, event-based PM tasks such as “when I show you my phone tell me to put it on silent”, or “when I show you a red pen, sign your name” constitute explicit cues for PM retrieval presented in the focus of participant attention. In contrast, in the House PM task, individuals need to remember to press relevant room icons to switch attention between rooms to monitor for potentially presented event-based PM cues. Event-based PM in the House PM task therefore likely requires both self-initiated time- and event-based PM monitoring (Dismukes, 2010). The time monitoring demand associated with event-based PM were likely greater than for time-based PM, because event-based PM cues could have been presented at any point during the PM retention interval and were therefore not well-scaffolded by the passage of time (Huang et al., 2014; Waldum & Sahakyan, 2013).

The current outcomes highlight that the predictions of PM theories (Einstein et al., 2005; Scullin et al., 2010; Smith, 2003), that are developed based on empirical findings from experiments using highly abstract PM paradigms that present single (one-at-a-time) stimuli centred on a task display (Einstein & McDaniel, 1990) do not necessarily directly translate to more complex PM tasks that require shifting of attention between task stimuli to complete PM tasks. More recently, computational models of human cognition have been developed to formalise the cognitive control and capacity-sharing mechanisms underlying PM retrieval (e.g., Strickland et al., 2018, 2024), and notably, these models are also fitted to behavioural data derived from experiments presenting single (one-at-a-time) centralised stimuli.

Our newly developed House PM task can potentially be used to study a variety of PM research questions. For example, event- and time-based PM tasks could be placed in different experimental blocks to examine performance costs to ongoing tasks, which are indicative of differential capacity-demands of each PM task (Einstein et al., 2005; Hicks et al., 2005; Smith, 2003; but see Strickland et al., 2018). According to our interpretation of the current findings, there should be greater costs to the ongoing task caused by event- compared to time-based House PM tasks. The House PM task is also suited to studying the retrieval of PM tasks created by task interruptions (Wilson et al., 2018), the impact of PM retention intervals, task context (Smith, 2017), and the use of memory aids (Finstad et al., 2006). We argue that the House PM task has ecological validity, but we have not provided any evidence for this. It will be important for future research to examine whether House PM accuracy predicts variation in self-rated (or significant other rated) PM functioning, performance on naturalistic PM tasks, everyday functioning (e.g., activities of daily living) and/or health (e.g., medication adherence) (Rummel & Kvavilashvili, 2019). Finally, while some household PM tasks may have specific deadlines or cues, as in the current study, it will be important to study event- and time-based intentions with broader deadlines (e.g. I can successfully charge my computer, or move my laundry, if I remember to do so in a range of a few hours of time).

We conclude that the memory demand on the House PM task associated with event-based PM attention switching requirements was greater than the memory demand associated with time-based PM attention switching requirements, because event-based PM cues could be presented at any point during the PM retention interval and were not scaffolded by the passage of time (Huang et al., 2014; Waldum & Sahakyan, 2013). The current findings are important because PM demands in everyday life and in work settings can require individuals to not only to remember to perform a deferred task, but also to periodically remember to switch attention between different stimuli (Dismukes, 2012; Loft et al., 2014, 2019).

## Data Availability

The data is available upon request from the corresponding author (Shayne.Loft@uwa.edu.au).

## Declarations

The manuscript does not contain clinical studies or patient data.
